# Use of Fertility Control (Nicarbazin) in Barcelona: An Effective yet Respectful Method towards Animal Welfare for the Management of Conflictive Feral Pigeon Colonies

**DOI:** 10.3390/ani12070856

**Published:** 2022-03-29

**Authors:** Carlos González-Crespo, Santiago Lavín

**Affiliations:** Wildlife Ecology & Health Group and Servei d’ Ecopatologia de Fauna Salvatge (SEFaS), Departament de Medicina i Cirurgia Animals, Facultat de Veterinària, Universitat Autònoma de Barcelona (UAB), Bellaterra, 08193 Barcelona, Spain; santiago.lavin@uab.cat

**Keywords:** animal welfare, *Columba livia* var. *domestica*, control, ethics, management, nicarbazin, pigeon, reduction, urban

## Abstract

**Simple Summary:**

Feral pigeon is one of the most common urban species worldwide. Living free in urban areas, pigeons have found a favorable environment with the conditions to have a high fertility, thus leading to overcrowding, which causes thousands of millions in damages yearly. The management of overpopulated pigeon colonies presents numerous challenges, and several methods have been proposed and used. In recent decades, the concern in the society about animal welfare has grown, and now demands non-lethal methods in the management of wildlife, especially in cities. The management of feral pigeons in Barcelona, Spain, used to be carried out, like in many cities, by capture and elimination. However, as this method has been proven to be ineffective and the public concern about animal welfare has increased, in 2016, the Barcelona City Council decided to change the management towards a fertility control method. This study describes and evaluates, during three years, an efficient fertility control protocol that is able to reduce the number of pigeons in the treated colonies by 55.26% at the end of the study. The results of this study provide animal management administrations and companies with a non-lethal protocol to control pigeon populations while respecting animal welfare.

**Abstract:**

This study describes a three-year evaluation (2017–2019) of a fertility control protocol using nicarbazin (Ovistop^®^) to reduce the abundance of the most conflictive colonies of feral pigeon, *Columba livia* var. *domestica*, in Barcelona, Spain, as a long-term strategy based on animal welfare. The treatment was supplied to 34 pigeon colonies by automatic hopper feeders installed in public areas. A superiority study and a population monitoring study were carried out to evaluate differences in the abundance of the colonies, as well as the proportion of juveniles, the possible intake of nicarbazin by non-target species and the movement of individuals among colonies. The results showed statistical differences in the population trends between the test (−22.03%) and control (+12.86%) groups, and a significant steady decreasing trend in the pigeon abundance (−55.26%) was registered until the end of 2019. The proportion of juveniles was significatively lower in the test colonies, and a non-target species (Eurasian collared doves, *Streptopelia decaocto*) was observed consuming in a residual form. The protocol using nicarbazin is able to both control the abundance of pigeons, with no impact over non-target species, and respond to the public interest about animal welfare by providing an ethical method to manage overabundant and/or conflictive populations.

## 1. Introduction

Birds and mammals have been increasingly colonizing cities during recent decades in a process called synurbization [1], but other species, such as the feral pigeon (*Columba livia* var. *domestica*), started the process of colonizing the urban environment centuries ago. As other synurbic species in the urban environment, pigeons, due to the lack of predators and the more abundant food resources that are usually present in these environments, have high survival and reproductive rates, which lead to overpopulation and creates the conditions for the rise of human–wildlife interactions and associated conflicts [1,2,3,4]. Public health risks (i.e., *Chlamydophila psittaci*, *Cryptococcus neoformans* and *Salmonella* [3]) and infrastructural damages are the most common conflicts when dealing with a high density of pigeons in urban areas. The homogeneous environmental conditions of the urban ecosystem and the demographic dynamics maintain the stability of the colony’s demographics, unless external factors intervene [5].

Traditionally, the management of the pigeon population in the city of Barcelona was carried out by the capture and elimination of individuals in areas with high density and/or conflicts. Despite the fact that previous studies since 2009 [6] showed a lack of effectiveness of this method due to juvenile replacement resulting in an increase in the fertility of the colonies, to control the pigeon population in the city of Barcelona, the capture and elimination of pigeons continued as the sole management method until 2015, with a consequent waste of resources.

In recent years, there is a growing concern about animal welfare, especially in urban areas, where lethal methods are becoming commonly rejected, and, consequently, the general public interest in non-lethal methods, such as fertility control, is increasing [7,8,9]. Furthermore, the general public is increasingly demanding the use of non-lethal methods by the wildlife management administrations.

Due to the lack of effectiveness of the capture and elimination method and the increasing public concern towards animal welfare [7,8,9], the Barcelona City Council initiated a project to implement a fertility control method in 2016. This method was set out with the objective of reducing the number of pigeons in conflictive pigeon colonies, as well as to evaluate the effectiveness of the method. In addition, an awareness campaign was carried out for six months in 2018 and 2019, where citizen surveys were carried out to increase the knowledge about the feeding habits and motivations of the citizens of Barcelona, and those citizens who usually offer supplementary feeding, hereafter feeders, were asked to reduce the amount of food provided, as well as to avoid feeding the conflictive colonies.

Nicarbazin has proven to be a successful method that is respectful towards animal welfare in controlling not only certain conflictive pigeon colonies in historical areas in Italy [5,10], Spain [11] and USA [9,12], but also Canadian geese (Branta canadensis; [13]). The present study reports the initial years’ experience of reducing the abundance of pigeons in the colonies that are associated with a higher ratio of conflict on a big-city-scale. The aim of this study is (1) to describe the three-year experience using a fertility control protocol based on nicarbazin, to reduce the abundance of the most conflictive colonies in the city of Barcelona, Spain, as well as to evaluate both (2) the effectiveness of the veterinary medicine (Ovistop^®^, ACME srl, Cavriago, RE, Italy) to reduce the abundance of pigeons and (3) the protocol used as a management strategy in an attempt to establish a long-term strategy to control a worldwide-spread species in the urban ecosystem while respecting animal welfare.

## 2. Materials and Methods

### 2.1. Location of the Colonies

The first phase of the project, carried out between the months of November 2016 and January 2017, was a study to determine the most suitable pigeon colonies (PC) to be treated with NCZ. To locate the PCs, the 73 neighborhoods included in the ten districts of the city of Barcelona (Ciutat Vella, CIV; Eixample, EIX; Sants-Montjuïc, SMJ; Les Corts, LEC; Sarria-Sant Gervasi, SSG; Gràcia, GRA; Horta-Guinardó, HOG; Nou Barris, NOB; Sant Andreu, SAD; Sant Martí, SMT) were visited. A special effort was made during the visits in and around squares, parks, markets and churches, as well as in places where the City Council had received incidents from the citizens, as these were locations where PCs usually produced conflicts. The criteria for the selection and prioritization of the PCs were as follows: (1) the abundance and density of pigeons in the colony (>100 pigeons); (2) the degree of conflict in the area determined from the number of citizen incidents registered by the Barcelona City Council (>5 incidents associated with the colony in the previous year); (3) the damage caused in the area (e.g., historical buildings and monuments) and (4) the proximity to places with higher risk (e.g., food markets, schools, nursing homes and health care centers) reporting problems to the Barcelona City Council.

**Figure 1:**
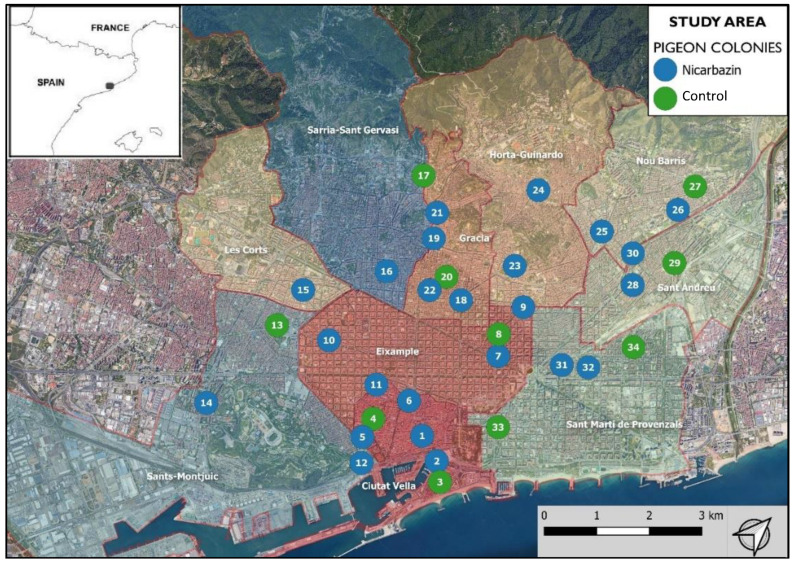
Study area. Location of the 34 conflictive colonies included in the study, divided in the treatment groups (test, nicarbazin; and control, control) of the 2017 superiority study.

As a result of the prioritization, of all the PCs in the city of Barcelona, the experiment included 34 PCs located in the ten districts of the city (Table 1, Figure 1).

### 2.2. Study Design

A three-year experimental model was designed for the evaluation of the efficacy of the contraceptive formulation on the urbanized population of feral pigeons, *Columba livia* var. *domestica*, in the city of Barcelona. As a veterinary medicine, the design of the study was performed accordingly to the guideline on statistical principles for clinical trials for veterinary medicinal products by the Committee for Medicinal Products for Veterinary use (CVMP) of the European Medicament Agency (EMA) [14].

The experiment was divided into three studies, (1) a superiority study (2017 treatment), (2) a palatability changes study (2017–2018 non-breeding period) with the aim to evaluate differences between test and control groups and (3) a population monitoring study (2017, 2018 and 2019 treatments) with the aim to evaluate differences in the abundance of each PC between treatment periods and years.

In the (1) superiority study (2017 treatment), 24 PCs colonies were randomly selected into test (treated with nicarbazin, NCZ, Ovistop^®^) and 10 PCs into control (treated with control, plain corn kernels) groups. The study design prioritizes some PCs treated with NCZ due to management issues of the project where the study was included, while making sure to include a PC treated with control in each of the 10 districts of the city.

The (2) palatability changes study (2017–2018 non-breeding period) was designed due to some concerns about a lower palatability of the product referred in laboratory conditions [15], which could cause a reduction in the number of pigeons baited in the automatic hopper feeders (AHFs). The study evaluated whether there were differences between the trends in the number of pigeons baited in AHFs with NCZ or control during the non-breeding period, and hence there was no recruitment of juveniles. The hypothesis was that a lower palatability of the product should cause a greater reduction in the total number of pigeons baiting on AHFs with NCZ compared with those with control. With the aim of balancing the total number of pigeons in each group, all of the PC colonies were ordered by their number of pigeons and alternatively included in each group (17 PCs into test (treated with NCZ, Ovistop^®^) and 17 PCs into control (treated with control, plain corn kernels) groups). The palatability changes study was not designed to evaluate effects on reproduction, as no effect was expected from treating pigeons with NCZ during the non-breeding period. Hence, no interactions between treatments were considered or analyzed.

In the (3) population monitoring study (2017, 2018 and 2019 treatments), all of the PCs included in the study were treated with NCZ.

#### 2.2.1. Census in the PC

To evaluate the efficacy of the antifertility method, the size of the PCs included in the study were estimated by means of census and monitored during the study period (2017–2019), and, then, the differences in the maximum number of pigeons in each period throughout the treatment (colony trend) were analyzed. Due to the behavior of attraction by the food and clustering, the period in which the maximum number of pigeons can be observed is at the moment of the treatment [5]. The data collection for the population estimates was carried out by means of repeated estimates (three days per period and colony) in three different periods of the year, pre-treatment (from 15 February to 15 March), treatment (from 15th to 31th of July) and post-treatment (from 1st to 30th of November). On each day of data collection, three estimates were collected with two photographs for each estimate (i.e., 15 min before distribution, at the time of distribution and 15 min after distribution), of which, the estimate with the maximum number of individuals was recorded as the abundance value of the PC for that period. For the statistical analyses, the data used were the higher number of pigeons in each PC collected in the pre-treatment and post-treatment censuses. These results were also used to ensure the availability of food for the entire PC by modifying the dose administered according to the number of pigeons in the colony.

#### 2.2.2. Juvenile Proportion in the PCs

During the post-treatment census of the 2017 superiority study, the proportion of juveniles in 18 colonies, 13 colonies included in the test group and 5 colonies included in the control group, were monitored as an indicator of the effectivity of NCZ [10]. Juveniles were distinguished from adults using cere and iris color, which permitted discriminating juveniles at a distance [16].

#### 2.2.3. Pigeon Mobility between PCs

To evaluate if pigeons from neighboring PCs mingle or remain as independent colony, several adult pigeons were marked with colored leg rings in three PCs. In PC19 (yellow rings) and PC21 (blue rings), located 450 m away, 18 pigeons were marked in each. Finally, 16 pigeons in the PC32 were also marked (red rings), and potential presence of these pigeons was evaluated in PC31, located 500 m away. Nine days of observations of leg-ring-marked pigeons were made throughout the year 2018 during the pigeon censuses in the pre-treatment, treatment and post-treatment periods.

#### 2.2.4. Evaluation of the Intake of NCZ by Non-Target Species

During all of the population censuses carried out during the study period, a significant effort was made to visually evaluate the possible intake of NCZ by non-target species at the time of product administration. Moreover, six colonies were selected to be monitored at least one time every week during three months. The selection of those colonies was performed on the basis of their location inside urban green areas with high biodiversity [17].

### 2.3. The Protocol

#### 2.3.1. The Product

Nicarbazin (NCZ) is a veterinary medicine included in the group of carbanilides, belonging to the anticoccidials that has long been used globally to control coccidiosis in broiler chickens. It is an equimolecular complex of 4,4’dinitrocarbanilide (DNC) and 2-hydroxy-4,6-dimethylpyrimidine (HDP). DNC is the active component and HDP avoids the aggregation of DNC, allowing for the absorption in the intestine [18]. When laying or breeding hens ingest nicarbazin, it causes reductions in egg laying and hatchability [13,19,20,21], and birds recover fully 4–6 days after being taken off treated food [22]. At appropriate dosages for anti-fertility treatment, nicarbazin exclusively affects the processes associated with the maturation of the egg; as a result, the product does not interfere with physiological processes, including those related to the reproductive apparatus [23].

The product used in the protocol for the control of pigeon colonies is a veterinary medicine registered in Europe, Italy (trade name Ovistop^®^) and Belgium (trade name R12^®^, ACME srl, Cavriago, RE, Italy), to reduce the fertility of feral pigeons, whereas, in the United States, nicarbazin is used for Canadian geese and feral pigeons. The active ingredient of the veterinary medicine registered in Europe and used in the present study (Ovistop^®^) is NCZ at 800 mg/kg, mixed with corn kernels and protected by stearic acid, BHT (butylated hydroxytoluene) and dimethicone.

#### 2.3.2. Method of Administration

**Figure 2:**
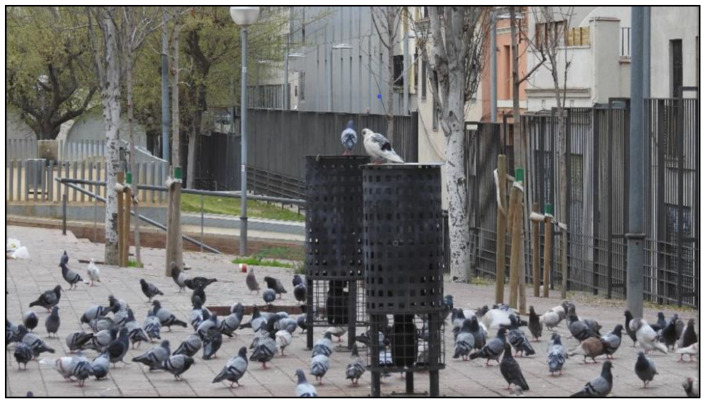
Automatic hopper feeders used for the administration of the dose.

The treatment was supplied to the pigeons by means of an automatic hopper feeder (AHF, Figure 2) installed on public areas, such as streets and parks. The AHF had a capacity of 30 kg and was covered by a case assembling the design of the street bin furniture used in the city of Barcelona. The control unit activated the motor, administrating and dispersing the dose at approximately five meters around at the scheduled time. The technical capabilities of the AHF allowed for the administration of a maximum dose for 110 pigeons. Several PCs in the study were high-density colonies with an abundance ranging from 120–220 individuals; therefore, two AHFs were installed in these locations to administer the recommended dose.

#### 2.3.3. The Treatment

The treatment consisted of a single daily distribution of the product at each point of treatment. The quantity of corn (Ovistop^®^ or control) given on a daily basis to each pigeon was 10 g, the dose indicated by the manufacturer. The total dose administered by each AHF was defined in the beginning of the study (March 2017). During a 15-day period, plain corn was administered to bait and maintain the attraction of the pigeons to the AHF. The total dose was periodically updated on the basis of the census carried out in each AHF. All AHF were programmed to simultaneously administer the dose at a specific time, allowing for a greater distribution of the dose within the population and therefore avoiding movement and feeding between different AHF by the same individuals.

The treatment period was designed to be running every year for nine months, from 15 February to 15 November, but due to factors not related to the project, the beginning of the administration was delayed in 2017 and 2019 after 15 March and 1 April, respectively. The dose was administered at 8:00 AM (from February to May) and at 7:00 AM (from June to November). The schedule was chosen as it was during one of the maximum concentrations of pigeons, as well as the fact that it the time of day where the possibility that a pigeon has obtained the food necessary for its daily requirements is minimal, so the intake of the daily dose of nicarbazin is ensured. The difference in the administration schedule depending on the month is a consequence of the time change during the summer period. To support the attraction of the pigeons to the AHFs between November and February, the non-breeding period, all AHFs administered 15% of the recommended dose.

Due to budget restrictions during the 2019 treatment, four PC (PC6, PC17, PC22 and PC28) composed of 20 or less individuals during the 2018 post-treatment census were removed from the study.

### 2.4. Analyses

Differences in the number of pigeons in each PC and proportion of juveniles between test and control groups, as well as differences in the number of pigeons in each PC among periods, were analyzed by means of linear mixed models (LMMs). The response variables were tested for normality and transformed if the normality assumption was not fulfilled.

Due to street works nearby the AHFs, the data obtained during the 2017 post-treatment census (Table 1) in PC4 (60 pigeons) and PC24 (102 pigeons) did not reflect the real number of pigeons in the colonies, as an important part of the colonies stopped being baited in the areas where the AHFs were located. Therefore, both colonies were discarded from the 2017 superiority study analysis.

In the superiority study of the 2017 treatment, no significant differences (F_1_ = 0.8757 *p* = 0.3564) were found in the initial abundance of pigeons between the treatment (117.75 ± 11.32 SE) and the control (97.5 ± 19.73 SE) groups; however, due to the differences in the initial number of pigeons in each colony included in the study (Table 1), the response variable used was the percentage difference between the number of pigeons in the pre-treatment and post-treatment censuses (change). In the palatability changes study (2017-2018 non-breeding period), the response variable used was the difference in the number of pigeons in each PC between the 2017 post-treatment and 2018 pre-treatment censuses. In the analysis of juveniles between test and control groups, the proportion of juveniles in each colony was transformed by means of a log transformation and used as response variable. In the three aforementioned LMMs, group was included as fixed component and district was included as random component.

In the population monitoring study, the higher number of pigeons in each PC collected during the 2017, 2018 and 2019 pre-treatment and post-treatment censuses was used as response variable after applying a square root transformation. Period (2017, 2018 and 2019 pre-treatment and post-treatment) was included as fixed component and PC and district were included as nested random components. Individual differences among all periods were analyzed by means of Tukey’s post hoc test with holm adjustment.

Statistical analyses on population trends of the PCs included in the study were performed using R 3.4.3 (R Core Team, 2017). LMMs were made with the lme4 package developed by [24], and post hoc tests for the LMM were made using the multcomp package [25].

## 3. Results

### 3.1. 2017 Superiority Study

**Figure 3:**
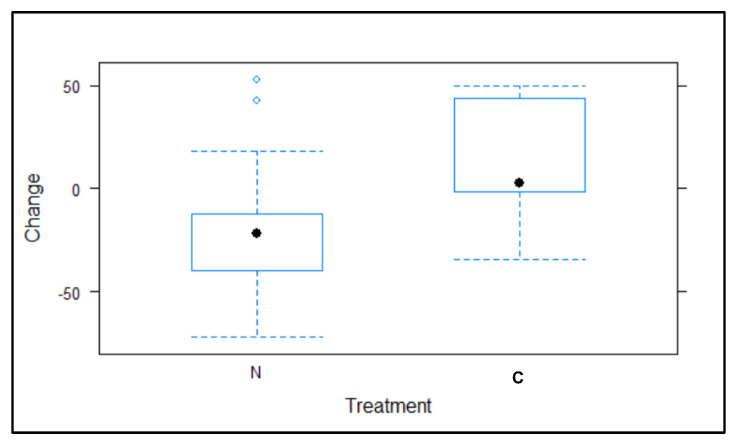
Percentage difference (change), between the 2017 pre-treatment and post-treatment periods, according to the treatment group, test (N, nicarbazin) and control (C)).

The results of the 2017 superiority study showed statistical differences between the test and control groups at the end of the treatment (*t* = 2.909, *p* = 0.00677, Figure 3).

**Figure 4:**
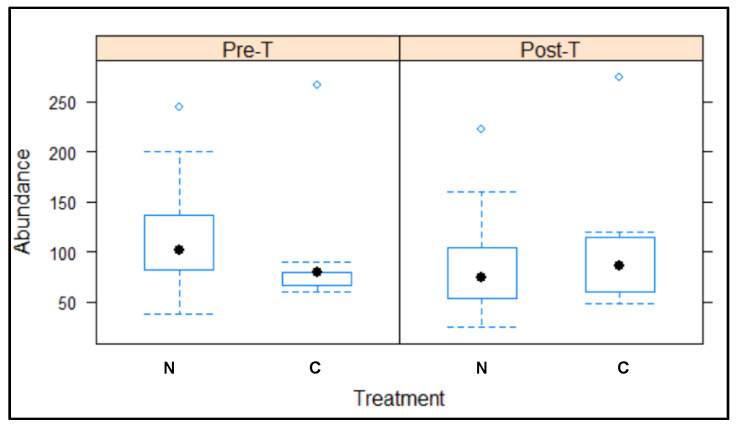
Differences in the average abundance of pigeons in each colony included as test (N, nicarbazin) and control (C) groups between the 2017 pre-treatment (Pre-T) and post-treatment (Post-T) periods.

**Figure 5:**
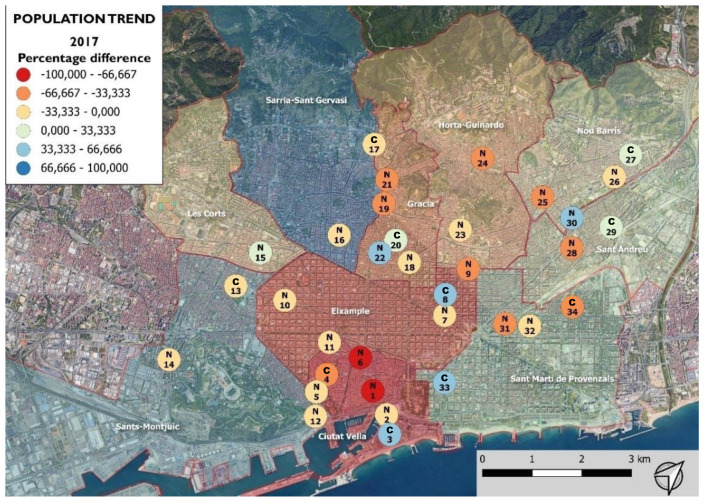
Heat map of the population trends registered during the 2017 superiority study (2017 pre-treatment and post-treatment periods) in the pigeon colonies included as test (N, nicarbazin) and control (P, control) groups.

In the population trend registered between the beginning and end of the 2017 treatment (Figure 4 and Figure 5), the number of pigeons in the 23 colonies treated with NCZ (excluding PC24) decreased by −22.03% ± 6.37 SE (−626 pigeons). Meanwhile, the number of pigeons in the nine colonies treated with the control (excluding PC4) increased by 12.86% ± 10.14 SE (94 pigeons).

#### Proportion of Juveniles

The same trend is registered in the analysis of the proportion of juveniles in the 18 colonies analyzed (Table 2). The results showed that, at the end of the 2017 treatment, the proportion of juveniles in the colonies included in the test group (10.93% ± 0.85 SE) was significantly lower (*t* = 8.128, *p* = 6.30 × 10^−7^) than the control group (36.20% ± 2.97 SE).

### 3.2. Palatability Changes Study (Non-Breeding Period 2017 Post-Treatment—2018 Pre-Treatment)

**Figure 6:**
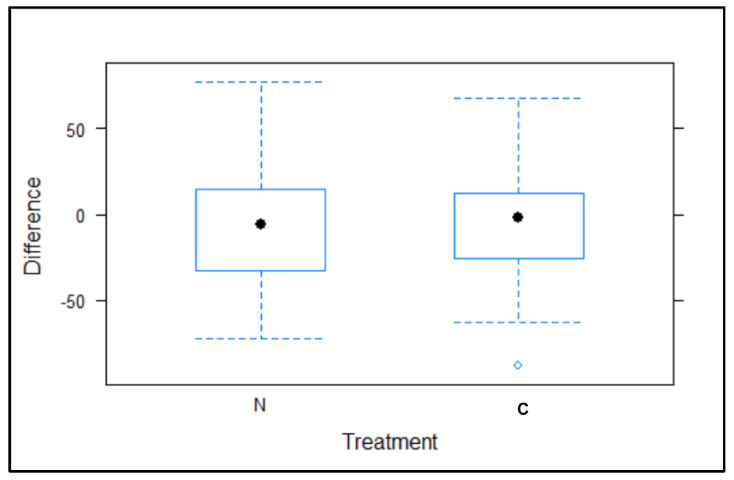
Difference in the number of pigeons between 2017 post-treatment and 2018 pre-treatment periods, according to the test (N, nicarbazin) and control (C, control) groups.

The analysis of the differences in the number of pigeons feeding on AHFs with NCZ compared with the control during the non-breeding period (2017–2018), showed no statistical differences (*t* = 0.014, *p* = 0.9890, Figure 6) in the AHFs providing NCZ or control.

### 3.3. 2017–2019 Population Monitoring Study

**Figure 7:**
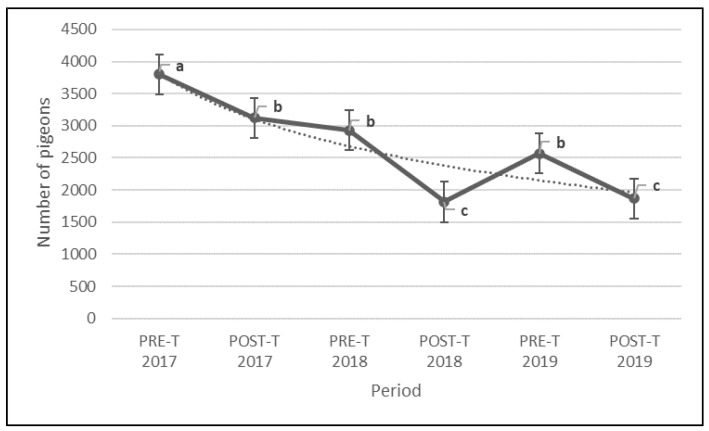
Population trend of the treated pigeon colonies during pre-treatment (PRE-T) and post-treatment (POST-T) periods of the study. Vertical lines indicate standard error; treatment periods with different superscripts (a, b, c) are different (*p* < 0.05).

**Figure 8:**
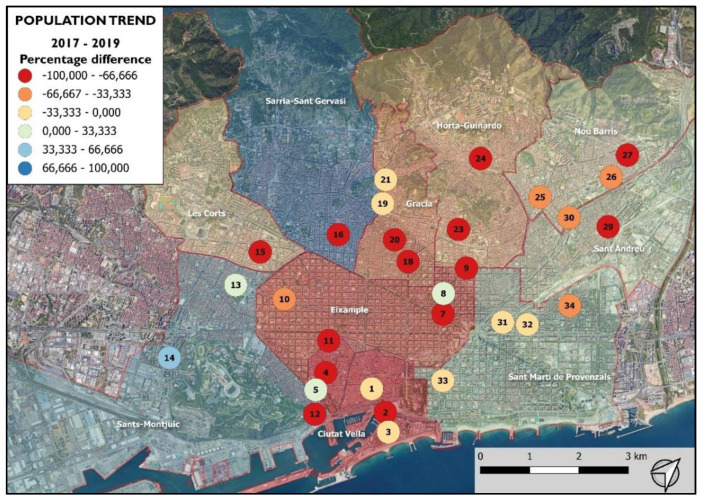
Heat map of the population trends registered in the pigeon colonies throughout the study period, from 2017 pre-treatment to 2019 post-treatment.

The results of the population monitoring study showed statistical differences (F = 24.724 *p* = < 2.2 × 10^−16^) in the number of pigeons of the PCs included in the study among the monitoring periods (Figure 7). From the beginning of the treatment (2017 pre-treatment period), a significant steady decreasing trend (Table 3) in the pigeon abundance was registered (Table 1). The results showed that the periods can be grouped into three clusters (a: 2017 PreT; b: 2017 PostT, 2018 PreT and 2019 PreT; and c: 2018 PostT and 2019 PostT), achieving a −55.26% average reduction (Figure 8) in the pigeon population (−1936 pigeons) throughout the study period (from 2017 pre-treatment to 2019 post-treatment).

A similar significant decreasing trend was captured by the analysis of the population trends between the pre-treatment and post-treatment periods for both the 2018 and 2019 treatments (Table 1 and Table 3), where the average reduction achieved between periods was −31,62% of the population (−1115 pigeons) during 2018 (Figure S1) and −26.16% of the population (−706 pigeons) during 2019 (Figure S2).

However, an increasing trend was registered during the 2018–2019 non-breeding period (Table 1 and Table 3), where the pigeon population increased by 29.93% on average (757 pigeons). As a result, the findings of the analysis of the population trends between pre-treatment or post-treatment periods among years (Table 1 and Table 3) showed a significant decreasing trend for 2017–2018 and 2017–2019 in both pre-treatment (2017–2018, −27.41%, −872 pigeons; 2017–2019, −29.1%, −1230 pigeons) and post-treatment (2017-2018, −37.59%, −1308 pigeons; 2017–2019, −33.82%, −1257 pigeon) periods, but not for the 2018–2019 pre-treatment and post-treatment periods.

#### 3.3.1. Pigeon Mobility between Colonies

During the study period, nine observation days of leg-ring-marked pigeons were carried out during the three 2018 census periods. No presence of marked pigeons was observed in neighboring PCs throughout the year 2018. All marked pigeons remained within their colony.

#### 3.3.2. Intake of NCZ by Non-Target Species

During the study period, 678 evaluations of possible consumptions of NCZ by non-target species were carried out. The consumption by collared doves and quantity of product consumed was evaluated by visual observations during the censuses around the AHFs at the time of the product administration Although the presence of other birds in the area was documented, no consumption of product by other species was recorded in the AHFs, except in the case of the Eurasian collared doves (*Streptopelia decaocto*). Eurasian collared doves were seldom observed as baited in seven PCs (PC5, PC11 and PC24 in the test group and PC4, PC8, PC29 and PC34 in the control group) during 2017 censuses, and in two PCs (PC5 and PC31) during 2018. Only PC5 registered collared doves baited in the AHFs in more than one census, and the consumption by this species was not recorded during 2019. The average quantity consumed by the collared doves was below six corn kernels (2–3 g).

## 4. Discussion

The protocol followed during the study has proven to not only be effective in the objective of reducing the number of pigeons in the colonies treated with nicarbazin, but also in respecting the welfare of the treated animals and non-target species. The implementation by wildlife management administrations in urban settings of efficient non-lethal management strategies, using NCZ to control the fertility in the most conflictive colonies, is recommended, as it has proven to be effective while, at the same time, responding to the general public increasing interest [7,8,9].

The results of the 2017 superiority study indicated a lower reproduction rate of pigeons in the colonies in the test group (treated with nicarbazin), with a consequent decrease in the recruitment of juveniles and, as a result, a decrease in the number of pigeons in the colony. The reduction achieved during 2017 in the test group agreed with previous experiences using NCZ, where the average reduction reached during the first year is 20–30% [5,10], presenting a significantly different population trend than the control group. In those colonies, the reproduction and, consequently, the recruitment of juveniles has continued at usual rates, therefore maintaining an increasing trend in the number of pigeons.

The increasing trend that was registered in the control group corresponds with the increasing trend obtained from the pigeon surveys that were conducted in the city of Barcelona when no control strategy was in effect (2015 survey: 85,777+/−10,028 pigeons; 2017 survey: 103,226+/−14,353 pigeons [26]). Despite the criticism of the survey method, due to its estimation of the pigeon population in a city as a whole instead of independent colonies, the use of controversial indexes and high confidence intervals (15% CI provided) [27,28], the results were considered as supportive information for the study and as an index of relative abundance.

The results of the three-year population monitoring study clearly describe a significant decreasing population trend throughout the study period. The protocol followed was effective in halving the total population in the colonies included in the study. The findings of this study are in agreement with previous experiences, where similar trends were described in experiences controlling pigeon colonies using NCZ in Italy [5,10], Spain [11], USA [9] and in Canada geese in USA [13].

In Mediterranean regions, the breeding season is long and can last almost all year, with a spring-summer peak, and the contribution of winter breeding attempts toward the yearly number of fledglings can be up to 41% [4,15]. In 2019, besides the treatment being delayed until April 1st, winter (2018–2019) and spring were unusually mild. The favorable meteorological conditions probably affected the reproductive success of feral pigeons. The aforementioned increasing trend during the 2018–2019 non-breeding period influenced the initial population during the 2019 treatment and, therefore, impacted not only the results of the analysis of the population trend during pre-treatment or post-treatment periods between years, but also the overall results after three years. Hence, in order to increase the effectiveness of the proposed method, it is recommended to implement the method as soon as the reproductive behavior is displayed, which, in areas with a Mediterranean climate, can be as soon as February.

The results of the mobility test, where all the marked pigeons remained in their PC, showed that each of the colonies act as an independent unit without the mixing of individuals. Using other indicators, such as radius censuses around the AHF, would require prior demonstration that all pigeons counted within the census radius belonged to the treated colonies. As the results showed, there is not a single population of pigeons but several independent groups (colonies) with defined movements towards feeding points. Thus, some pigeons within the radius of the census may belong to different colonies feeding in other locations rather than the AHF, i.e., not receiving treatment. Therefore, those pigeons were excluded from the experiment and are not a comparable sample.

The protocol described in the present study has proven to be effective in the objective of not affecting other non-target species. NCZ was selected for the present study due to its pharmacokinetic characteristics, posing minimal impact on treated birds, non-target species or the environment [29]. To produce any toxic effects, non-target mammals (including humans) would have to consume large amounts of the product. Based on the rat acute oral LD50 toxicology data to cause lethal effects in 50% of the population, an acute single ingestion for a 15 kg child or a 10 kg dog would have to exceed 60 kg and 40 kg of bait, respectively [29]. Despite that, a special effort was made in the design of the described protocol to minimize the time that NCZ was available for consumption, which was, on average, 10 s, to avoid consumption by non-target species. As a result, only some Eurasian collared doves rarely consumed a fraction of the required dose of NCZ (10 g of product), and not enough to cause any effect on reproduction, but only enough to cause an anticoccidial effect [19,20,22,23].

A previously published study [30] was performed to evaluate the effect of the treatment in areas surrounding the AHFs after one year of treatment. The interpretation of the results by the authors, stating no effect was registered, may not be appropriate, as their results indicated a different trend and statistical differences in the number of pigeons due to the treatment between test and control areas. However, the authors of the aforementioned study did not follow the EMA guidelines principles [14] in their methodology, even though they use a part of the data from the present study. As established in the EMA guidelines [14], comparison with external controls has been avoided in our study as it may lead to erroneous conclusions due to the fact that they differ from the test group in more factors than just the treatment. In this study, Barcelona was used, which is a dense urban area with a great variation in its urban fabric and urban spaces. Furthermore, the comparison with external controls should also be avoided due to the existence of the magnet effect [5] to the areas with AHFs. When a stable and daily food source emerged, pigeons are attracted to it, therefore generating an abundance increase in the PCs around them. However, as a result of not having this stable food source, the attraction to nearby areas lacks external control.

The results of the palatability changes study (2017–2018 non-breeding period) (Table 3, Figure 6) showed that, during the non-breeding season, there are no differences in the trend of the number of pigeons feeding daily in the AHFs with nicarbazin or the control. Both groups registered the same decrease in the number of pigeons feeding at the AHFs, which is related with natural mortality during winter. The findings of the current study, such as the general increasing trend registered during the 2018–2019 non-breeding period and the increasing trends registered in some PCs, can suggest a maintained interest towards the AHFs.

**Figure 9:**
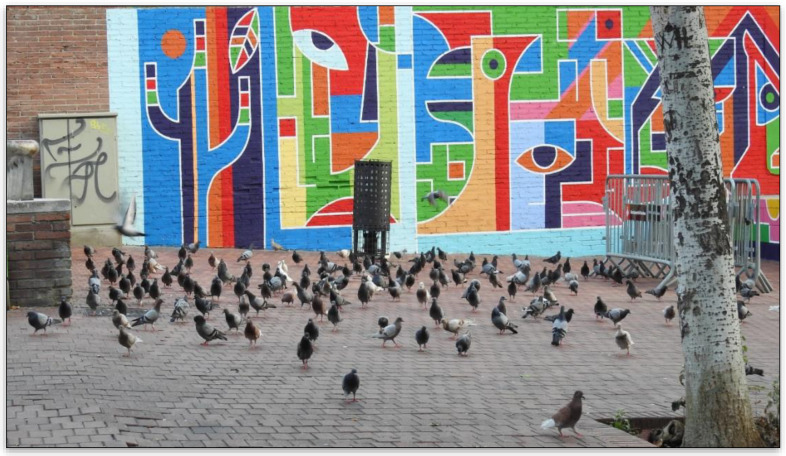
Pigeons waiting around an AHF for the administration of the dose.

A behavior displayed by the pigeons and registered during the census throughout the study period was that, 15 min before the time of the daily administration, the pigeons had already gathered around the AHF, and, at the time of the administration of the treatment (Ovistop^®^), struggles had been established to access the grains (Figure 9, Video S1). This behavior indicates eagerness for food, and none of the observations showed any unconsumed product. According to previous studies [31], in these struggles, the adult individuals are always at the forefront due to their greater aggression.

This behavior may also indicate an increased attraction and fidelity of the pigeons to the AHFs, compared to manual administration by operators [5,11]. The characteristics provided by the AHFs, such as the automatic distribution of the product in the same location and time during the entire treatment period, seemed to enhance the attraction and fidelity of the group of pigeons in the colony, increasing the effectiveness of the treatment and reducing the differences in the results between different colonies. Another important practical implication is the cost of the fertility control method when compared with other methods to control pigeon abundance in urban areas. The cost of the product (Ovistop^®^) during the study period was, in total, EUR 387,143 (EUR 126,000 in 2017, EUR 144,755 in 2018 and EUR 116,388 in 2019). On the other hand, the average yearly cost of capture and elimination over the last five years carried out in Barcelona was EUR 90.000/year, which would have resulted in EUR 270,000 during the three years of the study period. While the expenses produced by the capture and elimination method must be maintained in the long term on a yearly basis, the economic expenses of implementing a fertility control method, such as nicarbazin, are reduced annually as the number of pigeons to be treated decreases.

The main limitation of the protocol used is the presence of feeders in the areas where the AHFs were located. These citizens interfere with the treatment by moving pigeons from and to surrounding areas and increasing the maximum abundance of pigeons in the colony. Several PCs included in the study were severely affected by the presence of feeders in different scenarios. During the 2017–2018 non-breeding period, the number of pigeons in PCs 1, 6,14, 21 and 26 were significantly increased. These PCs are located in areas strongly affected by the presence of feeders. PC5 is located in a park where dozens of citizens leave different quantities of food uninterruptedly throughout the day, whereas, in PC26, a single feeder provides the colony with approximately seven kilograms of food once a day.

Other limitation of this study is the lack of data about the effect of immigration in the dynamics of the colonies. Therefore, to improve the efficacy of the method, further research should be undertaken to investigate the impact of immigration in the population dynamics of the colonies and, in particular, the role of juveniles. In addition, further work is required to establish a better understanding of the daily movements of pigeons in urban areas and to ascertain the presence of the same individuals baited in each AHF.

## 5. Conclusions

Overall, the present study describes an efficient protocol that, by means of the use of NCZ, is able to (1) control and reduce the abundance of pigeons in treated colonies, (2) have no impact over non-target species and (3) not only avoid the growing antipathy toward lethal methods from the society but respond to the public interest about animal welfare by providing an ethical method to manage overabundant and/or conflictive populations [7,8].

For that reason, the most efficient management strategy aimed to control conflictive pigeon colonies while respecting animal welfare should include (1) the study of the pigeon incidents recorded by the City Council, which has proven to be a useful data source for the location of conflictive colonies and nesting areas, (2) citizen surveys addressed to increase the knowledge about the motivations of the citizens for feeding and, elucidated by the findings of (1) and (2), (3) fertility control over the conflictive colonies in order to reduce the abundance of pigeons, (4) a reduction in the nesting areas [4] and (5) awareness campaigns based on the motivations of the feeders in order to reduce supplementary feeding.

## Figures and Tables

**Table 1:** Scheme of the study design and maximum number of pigeons recorded in the pre-treatment (PreT) and post-treatment (PostT) censuses during the study period. Group (N, nicarbazin; C, control). District (Ciutat Vella, CIV; Eixample, EIX; Sants-Montjuïc, SMJ; Les Corts, LEC; Sarria-Sant Gervasi, SSG; Gràcia, GRA; Horta-Guinardó, HOG; Nou Barris, NOB; Sant Andreu, SAD; Sant Martí, SMT).

Pigeon Colony	District	Group ^a^	Group ^b^	PreT 2017	PostT 2017	PreT 2018	PostT 2018	PreT 2019	PostT 2019
				Superiority study					
					Palatability changes study				
				Population monitoring study					
PC1	CIV	N	N	107	30	67	95	95	74
PC2	CIV	N	N	190	155	83	58	90	43
PC3	CIV	C	C	80	120	112	44	74	67
PC4	CIV	C	C	124	60	92	20	100	32
PC5	CIV	N	C	170	160	158	102	200	200
PC6	CIV	N	C	85	25	56	15		
PC7	EIX	N	C	150	123	89	40	80	47
PC8	EIX	C	C	80	115	95	65	116	105
PC9	EIX	N	N	112	56	56	20	36	20
PC10	EIX	N	N	130	105	68	22	75	59
PC11	EIX	N	N	102	75	69	59	81	25
PC12	SMJ	N	C	100	100	103	29	93	24
PC13	SMJ	C	N	61	60	51	40	72	67
PC14	SMJ	N	C	78	62	130	52	133	123
PC15	LEC	N	N	110	130	75	62		13
PC16	SSG	N	C	89	75	84	30	43	29
PC17	GRA	C	C	60	48	60	0		
PC18	GRA	N	N	56	39	30	21	20	11
PC19	GRA	N	C	78	50	82	21	41	53
PC20	GRA	C	N	67	68	35	19	24	20
PC21	GRA	N	N	93	48	74	68	80	72
PC22	GRA	N	C	38	58	60	20		
PC23	HGI	N	C	142	103	40	20	72	30
PC24	HGI	N	N	235	102	134	171	119	72
PC25	NOB	N	N	200	95	110	116	108	130
PC26	NOB	N	N	245	223	300	160	260	158
PC27	NOB	C	C	267	274	186	120	136	48
PC28	SAD	N	N	68	38	37	18		
PC29	SAD	C	C	67	86	65	44	93	15
PC30	SAD	N	C	52	74	48	35	50	20
PC31	SMT	N	C	105	70	40	50	60	100
PC32	SMT	N	N	91	71	67	35	57	89
PC33	SMT	C	N	79	115	76	99	90	65
PC34	SMT	C	N	90	59	37	24	47	31
	TOTAL			3801	3122	2929	1814	2571	1865
	AVERAGE			111.79 ± 9.84 SE	90.35 ± 9.06 SE	84.38 ± 8.93 SE	52.76 ± 7.17 SE	87.76 ± 9.31 SE	61.40 ± 8.39 SE

^a^ Treatment groups during the superiority study (2017). ^b^ Treatment groups during the palatability changes study (2017–2018).

**Table 2:** Proportion of juveniles at the end of the 2017 superiority study in 18 pigeon colonies. Group (N, nicarbazin; C, control).

Pigeon Colony	Juveniles (%)	Group
PC7	11.6	N
PC9	15	N
PC10	11	N
PC11	10	N
PC12	9.6	N
PC18	10	N
PC19	5	N
PC22	8.5	N
PC25	13	N
PC26	13	N
PC28	12.5	N
PC31	16	N
PC32	7	N
PC13	32	C
PC27	40	C
PC29	30	C
PC33	33	C
PC34	46	C

**Table 3:** Results of the post hoc tests (Tukey test) of the registered population trend among treatment periods.

	2017 PostT ^b^	2018 PreT ^b^	2018 PostT ^c^	2019 PreT ^b^	2019 PostT ^c^
2017 PreT ^a^	−1.1067 ± 0.3768 SE *z* = −2.937, *p* = **0.016568**	−1.4122 ± 0.3768 SE *z* = −3.748 *p* = **0.001071**	−3.5532 ± 0.3768 SE *z*= −9.430 *p* = < **2 × 10^−16^**	−1.5459 ± 0.3957 *z* = −3.907 *p* = **0.000655**	−3.2312 ± 0.3916 *z* = −8.252 *p* = **3.11 × 10^−15^**
2017 PostT ^b^		−0.3055 ± 0.3768 *z* = −0.811 *p* = 1.000000	−2.4465 ± 0.3768 *z* = −6.493 *p* = **1.10 × 10^−9^**	−0.4392 ± 0.3957 *z* = −1.110 *p* = 1.000000	−2.1245 ± 0.3916 *z* = −5.425 *p* = **6.36 × 10^−7^**
2018 PreT ^b^			−2.1410 ± 0.3768 *z* = −5.682 *p* = **1.60 × 10^−7^**	−0.1338 ± 0.3957 *z* = −0.338 *p* = 1.000000	−1.8191 ± 0.3916 *z* = −4.645 *p* = **3.05 × 10^−5^**
2018 PostT ^c^				2.0073 ± 0.3957 *z* = 5.072 *p* = **3.93 × 10^−6^**	0.3220 ± 0.3916 *z*= 0.822 *p* = 1.000000
2019 PreT ^b^					−1.6853 ± 0.4052 *z* = −4.159 *p* = **0.000256**

^a, b, c,^ treatment periods with different superscripts are different (*p* < 0.05). *p*-values marked with bold indicate statistically significant differences between the groups.

## Data Availability

All data used can be found in the present study.

## Supplementary Materials

The following supporting information can be downloaded at: https://www.mdpi.com/article/10.3390/ani12070856/s1, Figure S1: Heat map of the population trends registered between the 2018 pre-treatment and post-treatment periods in the pigeon colonies included in the study. Figure S2: Heat map of the population trends registered between the 2019 pre-treatment and post-treatment periods in the pigeon colonies included in the study. Video S1: Pigeons waiting around an AHF for the administration of the dose and struggles to access the grains.
