# Supplementary material for: Use of Fertility Control (Nicarbazin) in Barcelona: An Effective yet Respectful Method towards Animal Welfare for the Management of Conflictive Feral Pigeon Colonies
Source: Animals (Basel). 2022 Mar 29;12(7):856. doi: 10.3390/ani12070856 (PMC8997112; doi:10.3390/ani12070856)
Supplement: Supplementary file 1 [file animals-12-00856-s001.zip › Figures S1 and S2.pdf]

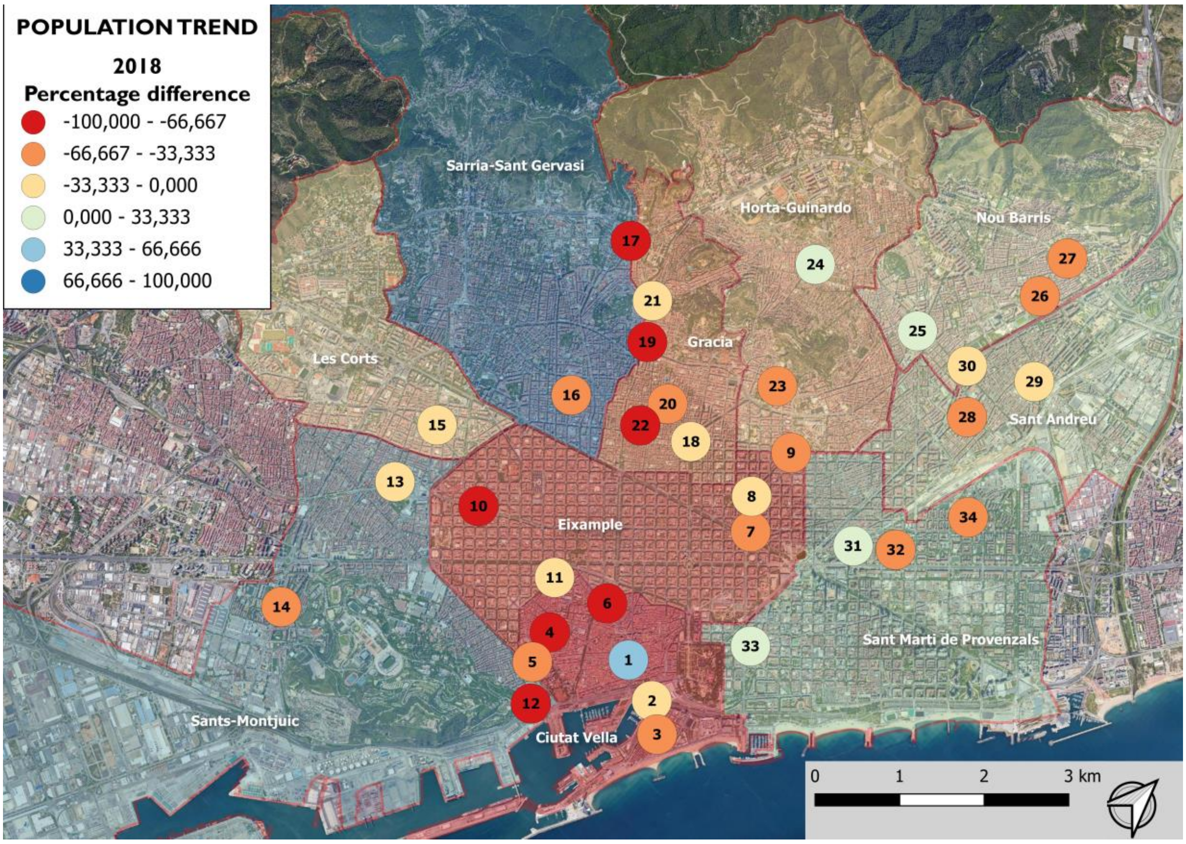

Figure S1. Heat map of the population trends registered between the 2018 pre-treatment and post-treatment periods in the pigeon colonies included in the study.

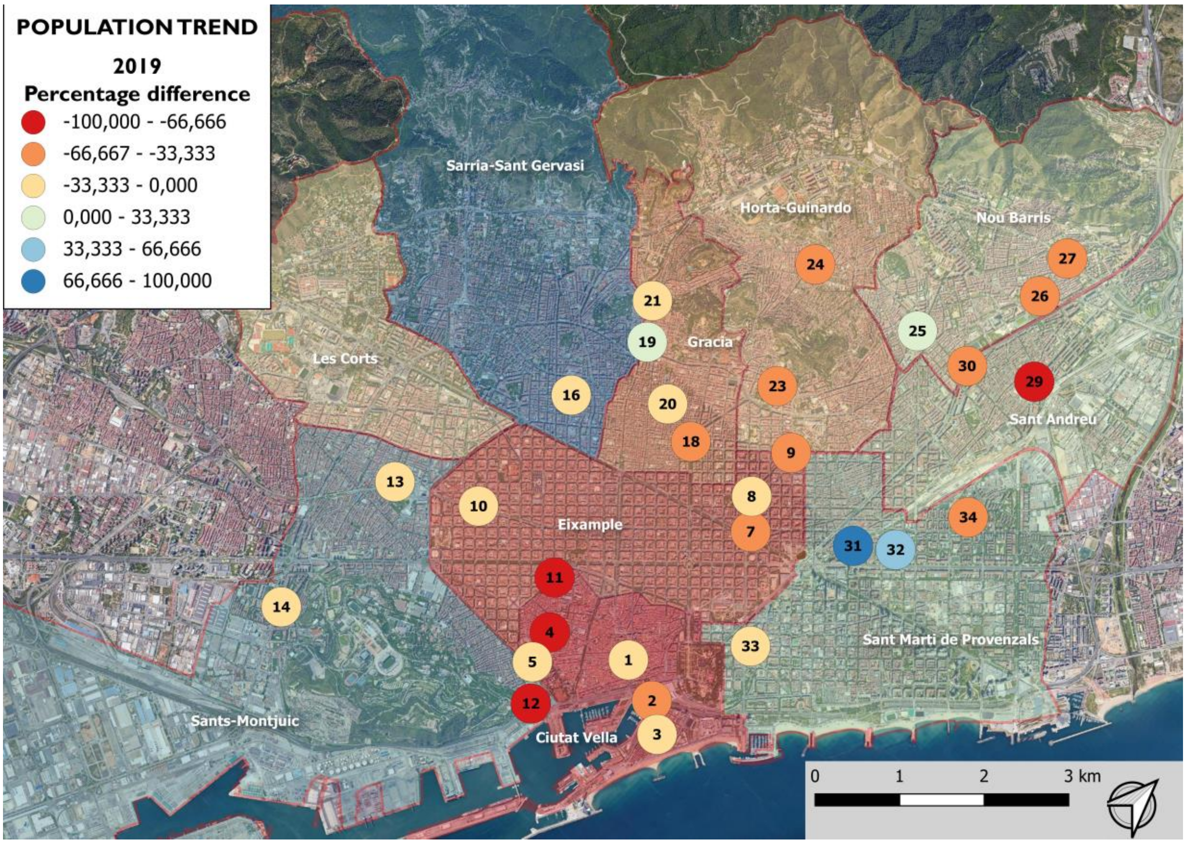

Figure S2. Heat map of the population trends registered between the 2019 pre-treatment and post-treatment periods in the pigeon colonies included in the study.
